# Circadian rhythm and epilepsy: a nationally representative cross-sectional study based on actigraphy data

**DOI:** 10.3389/fneur.2024.1496507

**Published:** 2024-12-03

**Authors:** Tianyou Tang, YuDong Zhou, Xuan Zhai

**Affiliations:** ^1^Department of Neurosurgery Children’s Hospital of Chongqing Medical University, Chongqing, China; ^2^Chongqing Key Laboratory of Child Neurodevelopment and Cognitive Disorders, Chongqing, China

**Keywords:** epilepsy, circadian rhythm, NHANES, cross-sectional study, actigraphy data

## Abstract

**Objective:**

The study aims to assess the relationship between epilepsy and circadian rhythms.

**Method:**

This study included a cohort of 7,410 participants sourced from the 2013–2014 National Health and Nutrition Examination Survey (NHANES) database. The investigation focused on the comparative analysis of seven nonparametric indices associated with circadian rhythms (Interdaily Stability (IS), Intradaily Variability (IV), Relative Amplitude (RA), L5, M10, L5 start time, and M10 start time) between the overall population and patients with epilepsy. Logistic regression analysis was utilized to assess the potential correlation between the rest-activity circadian rhythm patterns and the presence of epilepsy within the cohort.

**Results:**

Compared to the general population, individuals with epilepsy exhibited lower values of IS and M10. Multivariable logistic regression analysis, when IS, RA, and M10 were categorized into four groups based on quartiles, revealed that the odds ratio (IS: OR = 0.36, 95% CI: 0.13, 0.89; RA: OR = 0.25, 95% CI: 0.06, 0.77; M10: OR = 0.24, 95% CI: 0.06, 0.73) for the highest quartile was lower than that for the lowest quartile. Furthermore, after adjustment for confounding factors, participants in the highest quartile compared to those in the lowest quartile of IV and M10 start time demonstrated a higher prevalence of epilepsy.

**Conclusion:**

Individuals with epilepsy demonstrate significant alterations in circadian rhythms.

## Highlights

- We transformed the objective accelerometer data into circadian rhythm information.
- We conducted a cross-sectional study using the NHANES database to explore the association between circadian rhythms and epilepsy.
- Individuals with epilepsy exhibit significant changes in circadian rhythms.

## Introduction

Epilepsy is a severe neurological disease characterized by recurrent seizures, with a lifetime prevalence ranging from 1 to 3% (1, 2). The circadian rhythm is an intrinsic part of nearly all biological functions, exhibiting a 24-h daily cycle (3). The circadian rhythm of rest-activity is considered a reliable marker of the circadian system (4).

It has long been observed that epilepsy tends to manifest at specific times of the day. Consequently, researchers have endeavored to explore the relationship between the 24-h circadian rhythm and epilepsy. With advancements in technologies such as long-term electroencephalographic recordings, our understanding of the role of the circadian rhythm in epilepsy has been significantly enhanced (5, 6). However, existing studies have not clearly delineated the relationship between epilepsy and the circadian rhythm, and there is also a scarcity of clinical research based on objective data in this regard (7, 8). Liguori et al. included 22 patients with epilepsy and 17 controls, finding that patients with epilepsy exhibited lower synchrony and higher fragmentation of the rest-activity rhythm (9). This is currently the only study that has explored the association between the two using activity data. To address this gap in the literature, we leverage the robust data from the National Health and Nutrition Examination Survey (NHANES), a nationally representative cross-sectional survey database that contains detailed information on disease conditions and various lifestyle factors, thus demonstrating a high degree of generalizability. Specifically, this study quantified accelerometer data from the NHANES 2013–2014 dataset to measure the 24-h rest-activity rhythm in persons with epilepsy, aiming to yield novel insights into this critical area of research.

## Method

### Participants

**Figure 1:**
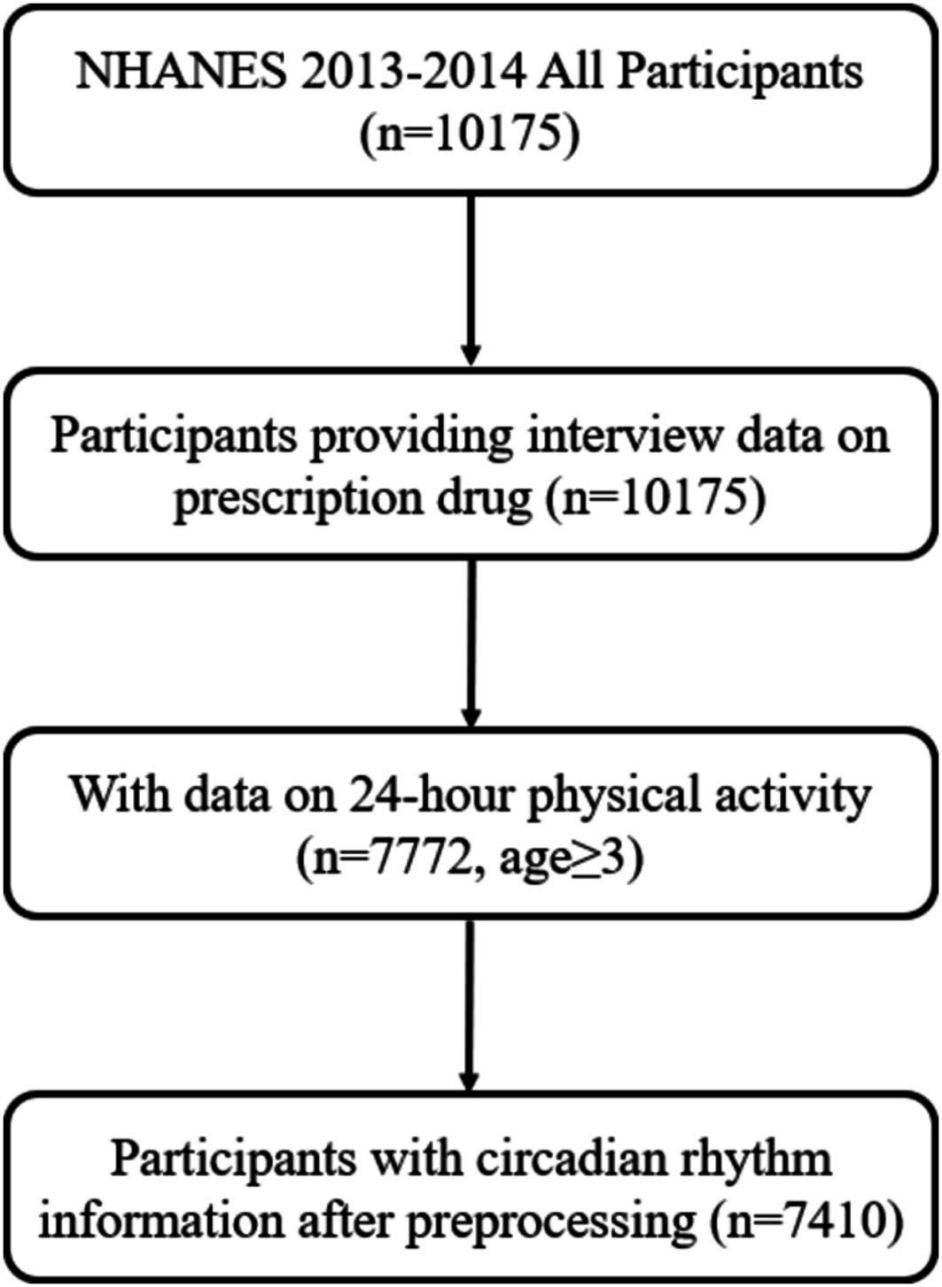
The inclusion of study participants.

Participants were sourced from the NHANES dataset. NHANES is a multi-stage, stratified, nationally representative cross-sectional study (10). The flowchart depicting the participant selection process is presented in Figure 1. Ultimately, a total of 7,410 participants were included. The NHANES protocol has been approved by the Research Ethics Review Board of the National Center for Health Statistics in the United States, and all participants provided informed consent.

### Data on rest–activity rhythm

Rest-activity rhythm data were collected from the Physical Activity Monitor (PAM) data. Participants in NHANES aged 3 years and older were instructed to wear ActiGraph GT3X+ devices continuously for seven consecutive days to collect objective data on 24-h movement patterns during both wakefulness and sleep. The PAM utilized in this study captured acceleration data on the x, y, and z axes at intervals of 1/80 s (80 Hz). Minute summary files and daily summary files were extracted as aggregated data. The triaxial values of the Microelectromechanical System Inertial Measurement Units (MIMS) at the minute level, denoted by the variable name PAXMTSM, were employed in the computation of the rest-activity rhythm. Accelerometer count values pertaining to a patient were designated as missing when PAXMTSM was recorded as “−0.01.” Additionally, if the device failed to continuously capture triaxial values, the accelerometer count values were also treated as missing. The procedures for accelerometer recording and data preprocessing have been outlined in previous studies (11, 12).

The R package “nparACT” was utilized to calculate the resultant nonparametric variables that characterize rest-activity rhythms, a methodology extensively elucidated in previous literature (13): (a) interdaily stability (IS), which gauges the fidelity of the rest-activity pattern in adherence to the light–dark cycle (IS ≃ 0 denotes resemblance to Gaussian noise, while IS ≃ 1 signifies optimal stability); (b) intradaily variability (IV), quantifying the extent of fragmentation (IV ≃ 0 represents a flawless sine wave, and IV ≃ 2 reflects Gaussian noise); (c) relative amplitude (RA), delineating the disparity between the peak activity period encompassing 10 consecutive hours (M10) and the trough activity period spanning 5 consecutive hours (L5) over an average 24-h period. (d) M10 start time denoting the initiation time of the peak activity phase; and (e) L5 start time offering insights into the commencement time of the nadir activity phase.

### The definition of epilepsy

The epilepsy-related information was derived from questionnaire dataset known as “prescription medications.” Participants self-reported their use of medications to treat “epilepsy and recurrent seizures” (International Classification of Diseases: G40). Furthermore, we manually screened the medications used by these participants, and those who were not taking standard antiseizure medications (ASM) were excluded from the analysis. Details are presented in Datasheet 1.

### Confounding variables

All confounding variables were discretized into categorical data. The demographic information was based on self-reported data, including age (3–19, 20–39, 40–59, > = 60 years), gender (female, male), race/ethnicity (Mexican, other Hispanic, non-Hispanic Black, non-Hispanic White, Other/multiracial), annual family income (<= $44,999, > = $45,000), and marriage status (never, married, separated). Body mass index (BMI) data was from examination data and classified into 0–18.5, 18.5–25, 25–30, >30 groups.

### Statistical analysis

We computed the unweighted frequencies and weighted proportions of categorical variables for both the epilepsy and non-epilepsy cohorts. Continuous variables were expressed as weighted means along with their corresponding 95% confidence intervals (CI). Subsequently, we employed multivariable logistic regression analysis using survey weights to ascertain the odds ratio (OR) of epilepsy with respect to rest-activity rhythm parameters. The strength of the association was evaluated using regression coefficients or ORs, accompanied by their respective 95% confidence intervals. We assessed three models with different sets of covariates. Model 1 did not involve any adjustments for additional covariates. Model 2 incorporated age, gender, and race as covariates. Model 3 further adjusted for annual family income and BMI. Statistical significance was defined as a *p*-value below 0.05. All statistical analyses were performed using the R software package.

## Result

### Baseline characteristics

All means (SD) and percentages were weighted based on the NHANES sampling frame. A total of 7,410 participants were included in the combined 2013–2014 NHANES sample. The mean age was 39.62 years (SD: 40.48), 52% were male, and 63% were non-Hispanic white. Among them, 53 individuals (0.75%) were identified as epilepsy patients. Detailed information can be found in Table 1.

**Table 1:** Characteristics of participants.

Variables	All (*N* = 7,410)
Age	
Mean (SD)	39.62 (40.48)
Median	32
Minimum	3
Maximum	80
Sex	
Female, *n* (%)	3,831 (48.01)
Male, *n* (%)	3,579 (52.00)
Race	
Non-Hispanic White	2,740 (63.03)
Non-Hispanic Black	1,651 (11.89)
Mexican American	1,261 (10.94)
Other/multiracial	1,081 (8.33)
Other Hispanic	677 (5.80)
Epilepsy	
No	7,357 (99.25)
Yes	53 (0.75)
Smoking status	
Current/ever smoker, *n* (%)	2075 (42.52)
Never smoking, *n* (%)	2,858 (57.48)
Physical activity	
Yes, *n* (%)	2,523 (45.73)
No, *n* (%)	3,277 (54.27)
Marital status	
Married, *n* (%)	4,312 (66.65)
Widowed, *n* (%)	429 (5.83)
Divorced, *n* (%)	828 (12.39)
Separated, *n* (%)	272 (2.69)
Never, *n* (%)	938 (12.445)
Income	
High, *n* (%)	1,265 (24.27)
Low, *n* (%)	5,870 (75.73)
BMI status	
Obesity, *n* (%)	1990 (31.85)
Normal weight, *n* (%)	2,312 (29.85)
Overweight, *n* (%)	1733 (27.25)
Underweight, *n* (%)	1,316 (11.05)
Rest-activity parameters	
IS, mean (SD)	0.52 (0.26)
IV, mean (SD)	0.76 (0.42)
RA, mean (SD)	0.70 (0.38)
L5, mean (SD)	2.24 (5.41)
L5 start time (min), mean (SD)	10:16:32 (968.24)
M10, mean (SD)	15.25 (7.53)
M10 start time (min), mean (SD)	09:46:11 (253.83)

% and mean (SD) were weight-adjusted. These values were obtained through weighted calculations using the “WTINT2YR,” and weighted calculations were performed using the “survey” package.

### Different rest-activity circadian rhythm

Differences in the circadian rest-activity rhythm indices between epilepsy patients and healthy controls are presented in Table 2, with weighted means and standard deviations. Compared to healthy participants, epilepsy patients exhibited a lower IS value (*p* < 0.05), indicating lower daytime stability in epilepsy patients compared to healthy individuals. Additionally, the M10 of epilepsy patients was significantly lower than that of normal individuals.

**Table 2:** Differences in circadian rhythms between individuals with epilepsy and the normal population.

Epilepsy	IS	IV	RA	L5	L5 start time	M10	M10 start time
Yes (*N* = 53)	0.50588 (0.2417)	0.72489 (0.2359)	0.73126 (0.2854)	2.11539 (3.320)	500.38 (689.5138)	12.81016 (4.9519)	594.41 (190.5860)
No (*N* = 7,357)	0.52045 (0.2659)	0.69927 (0.3688)	0.75541 (0.4374)	2.24140 (0.5667)	617.41 (982.7865)	15.27328 (7.7710)	586.12 (252.3526)
*p* value	0.03239	0.1613	0.3265	0.3593	0.9854	0.00129	0.47

Mean (SD) were weight-adjusted.

### Relationship between rest-activity circadian rhythm and epilepsy

We classified the circadian rhythm parameters based on quartiles and used logistic regression analysis to examine the relationship between different circadian rhythm parameters and epilepsy. In all models, individuals categorized in the highest quartile of IS, RA, and M10 exhibited a reduced prevalence of epilepsy in contrast to those classified in the lowest quartile. Additionally, following adjustments for all covariates, individuals falling into the highest quartile of IV and M10 start time demonstrated an elevated prevalence of epilepsy in comparison to those in the lowest quartile (IV: OR = 2.52, 95% CI: 1.04–7.09; M10 start time: OR = 3.13, 95% CI: 1.39–7.58) (see Table 3).

**Table 3:** Relationship between rest-activity circadian rhythm and epilepsy.

Rest-activity parameters	Model 1 OR (95%CI)	Model 2 OR (95%CI)	Model 3 OR (95%CI)
IS			
Q1	1	1	1
Q2	1.17 (0.63, 2.22)	1.08 (0.58, 2.06)	1.18 (0.61, 2.30)
Q3	0.36 (0.13, 0.87)	0.33 (0.12, 0.79)	0.36 (0.13, 0.89)
Q4	0.40 (0.15, 0.91)	0.38 (0.15, 0.88)	0.36 (0.13, 0.89)
P for trend	0.005	0.004	0.005
IV			
Q1	1	1	1
Q2	2.12 (0.88, 5.59)	2.02 (0.83, 5.35)	2.45 (0.97, 6.95)
Q3	1.76 (0.71, 4.74)	1.48 (0.59, 4.02)	1.64 (0.61, 4.80)
Q4	3.08 (1.36, 7.87)	2.25 (0.97, 5.86)	2.52 (1.04, 7.09)
P for trend	0.052	0.2	0.15
RA			
Q1	1	1	1
Q2	1.10 (0.59, 2.06)	0.98 (0.52, 1.85)	0.88 (0.46, 1.70)
Q3	0.47 (0.20, 1.02)	0.46 (0.20, 0.99)	0.48 (0.20, 1.06)
Q4	0.18 (0.04, 0.52)	0.22 (0.05, 0.65)	0.25 (0.06, 0.77)
P for trend	<0.001	0.008	0.041
L5			
Q1	1	1	1
Q2	1.35 (0.57, 3.32)	1.39 (0.58, 3.41)	1.22 (0.50, 3.05)
Q3	2.74 (1.32, 6.24)	2.62 (1.25, 6.02)	2.14 (1.00, 4.98)
Q4	0.90 (0.34, 2.36)	0.97 (0.36, 2.56)	0.93 (0.35, 2.48)
P for trend	0.01	0.023	0.11
L5 start time			
Q1	1	1	1
Q2	1.72 (0.83, 3.74)	1.79 (0.86, 3.92)	1.79 (0.84, 4.06)
Q3	0.99 (0.42, 2.31)	1.09 (0.46, 2.56)	1.08 (0.44, 2.65)
Q4	1.08 (0.47, 2.49)	1.10 (0.48, 2.55)	1.16 (0.50, 2.76)
P for trend	0.4	0.4	0.4
M10			
Q1	1	1	1
Q2	0.35 (0.17, 0.68)	0.38 (0.18, 0.76)	0.45 (0.21, 0.90)
Q3	0.22 (0.09, 0.48)	0.27 (0.10, 0.60)	0.33 (0.13, 0.74)
Q4	0.13 (0.04, 0.32)	0.18 (0.05, 0.51)	0.24 (0.06, 0.73)
P for trend	<0.001	<0.001	0.011
M10 start time			
Q1	1	1	1
Q2	1.30 (0.57, 3.05)	1.37 (0.60, 3.22)	1.55 (0.66, 3.76)
Q3	1.01 (0.41, 2.46)	1.50 (0.60, 3.74)	1.81 (0.71, 4.68)
Q4	2.03 (0.97, 4.53)	2.97 (1.37, 6.82)	3.13 (1.39, 7.58)
P for trend	0.2	0.037	0.051

Model 1: no additional adjustments. Model 2: adjusted for age, sex and race. Model 3: adjusted for age, sex, race, income and BMI.

**Figure 2:**
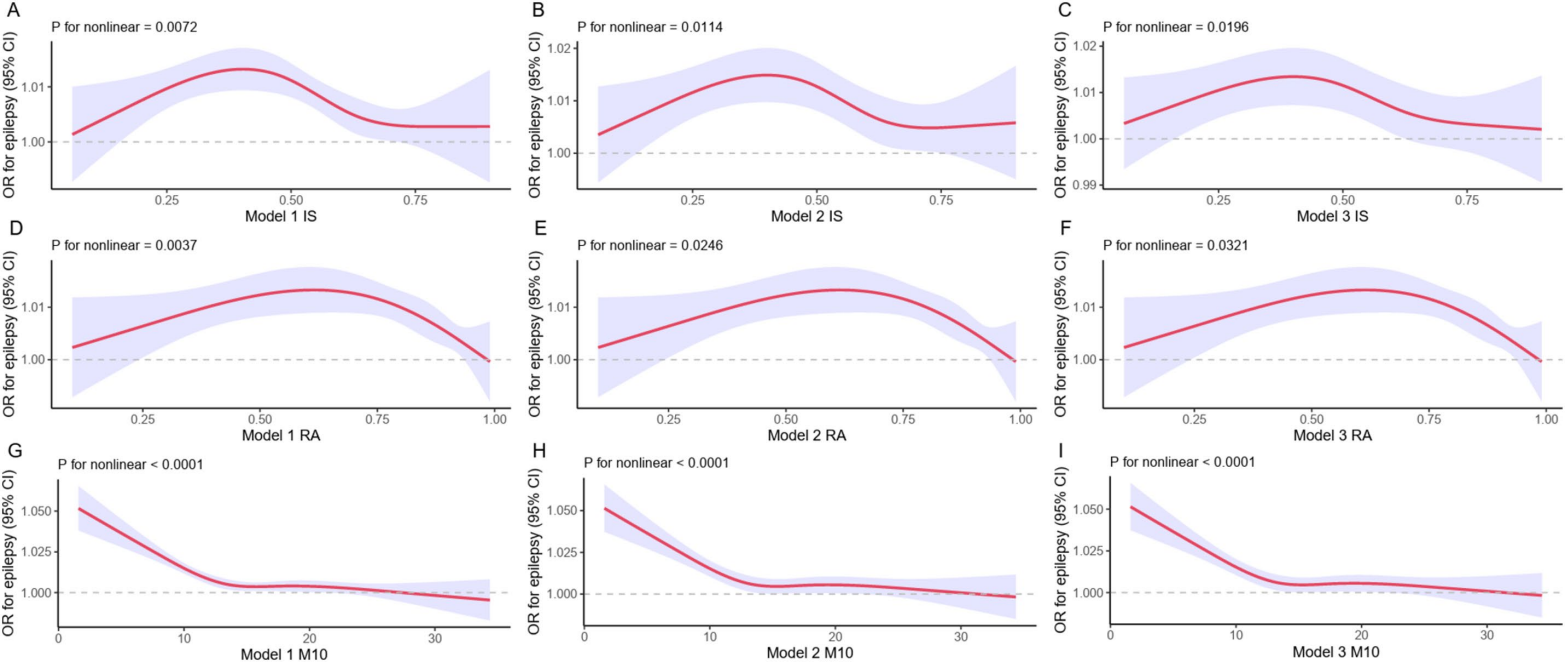
RCS models for epilepsy and rest-activity circadian rhythm. The RCS assessment evaluates the correlation of IS, RA, and M10 with epilepsy. The red solid line corresponds to the central estimate, while the purple shaded area represents the 95% confidence interval.

We further employed a restricted cubic spline (RCS) model to capture the nonlinear relationships between IS, RA, M10 and epilepsy. We observed that these relationships remained statistically significant (Figure 2). Although the outcomes of the nonlinear examination of the RCS exhibited minor discrepancies, the overarching patterns of the reliant and autonomous variables remained largely coherent throughout the diagrams.

## Discussion

Despite advances in EEG technology deepening our understanding of the association between circadian rhythms and the risk of epileptic seizures, there is a clear lack of clinical studies based on objective data. Previously, only one study utilized actigraphy to assess the relationship between rest-activity cycles and epilepsy (9). In contrast to this study, our cross-sectional study has a larger sample size, enabling more robust statistical analyses. Additionally, the NHANES database represents a wide spectrum of the U.S. population, while the study by Claudio Liguori et al. was based solely on patients visiting a healthcare center in Rome, potentially introducing selection bias.

Participants in NHANES aged 3 years and older were instructed to wear ActiGraph GT3X+ devices continuously for 7 days to collect information on 24-h movement during wakefulness and sleep. Actigraphy is considered the optimal tool for studying circadian rhythms and sleep (14). The current study represents the first comparison of circadian rhythms in individuals with epilepsy and the general population, providing the most comprehensive objective analysis of epilepsy and circadian rhythms to date. Through rigorous preprocessing and data transformation, we were able to directly identify differences in seven circadian rhythm parameters between individuals with epilepsy and the normal population. The significant differences identified were in IS and M10: individuals with epilepsy exhibited a more synchronized rest-activity rhythm with the light–dark cycle compared to the normal population, with increased daytime activity levels.

After multivariable logistic regression analysis, we found that Participants with greater IS were linked with a decreased prevalence of epilepsy, whereas individuals with elevated levels of IV displayed a converse relationship. These are all evidence of a close association between disruptions in the 24-h rest-activity circadian rhythm and epilepsy. Although the directionality of the associations in this cross-sectional study cannot be determined, existing evidence suggests a complex bidirectional relationship between circadian rhythms and epilepsy (15). Circadian rhythms are an inherent part of the biological physiological 24-h daily rhythm, influencing seizure occurrence and activity. This association may be related to the regulation of membrane excitability and neurotransmitter imbalance in neuronal circuits by circadian rhythms (16), as well as the involvement of core clock genes and circadian regulatory factors in influencing the expression of genes associated with epilepsy (17). During sleep, synchronous discharge of thalamocortical networks promotes the generation of non-REM sleep oscillations (18), while the circadian release of cortisol and melatonin may influence seizure occurrence (19). Studies indicate that the circadian rhythm system influences epilepsy susceptibility and seizure generation by regulating the timing of wakefulness and sleep stages and brain function (20). Sleep and wakefulness are considered the most reliable predictors of seizure occurrence, especially in temporal lobe epilepsy, where seizures are more frequent during sleep or exclusively occur during sleep. Furthermore, with increasing time awake, cortical excitability also increases, with a greater reduction in nighttime cortical excitability compared to daytime, potentially increasing the risk of seizures in the morning after sleep deprivation. Animal models also support the regulatory role of circadian rhythms in seizure occurrence, such as rodent models demonstrating endogenous circadian distribution of seizures (21, 22). These findings emphasize the significant impact of sleep–wake behaviors and circadian rhythms on epilepsy susceptibility and seizures, particularly in shaping the temporal patterns of seizures by regulating the timing of wakefulness and sleep stages and brain function. Therefore, understanding the circadian patterns of seizures and trends influenced by various factors, including epilepsy type, seizure onset region, and age, is crucial for predicting seizures, improving treatment, and ensuring patient safety.

Furthermore, our analysis revealed that participants in the highest quartile of M10, a variable indicative of the level of physical activity, exhibited a 76% reduced prevalence of epilepsy in contrast to those categorized in the lowest quartile. We believe this may be related to discouragement of physical exercise in individuals with epilepsy (23). In the past, exercise was thought to potentially trigger seizures by increasing stress, hyperventilation, liver enzyme metabolism, and exercise-related injuries (24). However, in reality, physical activity has no adverse effects for most patients, and animal models and cohort studies have confirmed that physical activity can improve epilepsy and its comorbidities (25, 26). Therefore, healthcare professionals should provide health education and encourage individuals with epilepsy to engage in safe physical activities.

Although this study has yielded exciting results, there are several limitations. Firstly, the type of epilepsy (generalized or focal) and the site of seizure onset (frontal or temporal lobe) play a role in the circadian epilepsy pattern. We identified individuals with epilepsy as those self-reporting seizures treated with standard ASM medication for epilepsy, and this information is not sufficient to determine the classification of epilepsy. Secondly, we utilized actigraphy data to generate indices of circadian rhythms. We were unable to verify the quality of the transformed data. However, rigorous preprocessing was conducted to ensure the quality of the seven indices. Thirdly, our raw data did not indicate specific timing of epileptic seizures, which hindered further elucidation of seizure patterns. Fourthly, the study did not specify whether participants were seizure-free while taking antiepileptic medications (ASM), which is crucial for understanding the relationship between circadian rhythms and seizure occurrence. Therefore, using ‘the intake of at least one antiepileptic medication (ASM)’ or the ICD code (G40) as proxy variables for identifying epilepsy may inherently introduce a potential risk of bias and limit the ability to draw conclusions about seizure control in this population. We advocate for more research based on objective data approaching from the perspective of circadian rhythms to offer new insights into predicting epilepsy.

## Data Availability

The original contributions presented in the study are included in the article/supplementary material, further inquiries can be directed to the corresponding author.
